# Supplementary material for: Sub-picosecond collapse of molecular polaritons to pure molecular transition in plasmonic photoswitch-nanoantennas
Source: Nat Commun. 2023 Jul 6;14:3875. doi: 10.1038/s41467-023-39413-5 (PMC10325968; doi:10.1038/s41467-023-39413-5)
Supplement: Supplementary file 1 — Supplementary Information [file 41467_2023_39413_MOESM1_ESM.pdf]

# Inventory of Supplementary Information


1. Supplementary Note 1. Sample fabrication.
   1.1. Supplementary Note 1.1 – Fabrication procedures
   1.2. Supplementary Note 1.2 – Determination of the molecular density and photoconversion yield
2. Supplementary Note 2. Computational part.
   2.1. Supplementary Note 2.1 – Quantum mechanical calculations of the isolated spiropyran and merocyanine molecules
   2.2. Supplementary Note 2.2 – Theoretical model to simulate the optical response of the coupled system
   2.3. Supplementary Note 2.3 – Theoretical discussion on the experimental results of Figure 5 (main text)
3. Supplementary Note 3. Time-resolved experiments.
   3.1. Supplementary Note 3.1 – Nonlinear optical response of Spiropyran and influence of photoisomerisation in pump-probe experiments
   3.2. Supplementary Note 3.2 – Determination of vibrational relaxation time scale
   3.3. Supplementary Note 3.3 – Ultrafast response of aluminum ellipse antennas
   3.4. Supplementary Note 3.4 – Fitting procedure of pump-probe time traces

SUPPLEMENTARY INFORMATION

# Sub-picosecond collapse of molecular polaritons to pure molecular transition in plasmonic photoswitch-nanoantennas

Joel Kuttruff[1¶], Marco Romanelli[2¶], Esteban Pedrueza-Villalmanzo[3,4¶], Jonas Allerbeck[1,5], Jacopo Fregoni[6], Valeria Saavedra-Becerril[4], Joakim Andréasson[4], Daniele Brida[7], Alexandre Dmitriev[3]*, Stefano Corni[2,8]*, and Nicolò Maccaferri[7,9,10]*

[1] Department of Physics, University of Konstanz, 78457, Konstanz, Germany
[2] Department of Chemical Sciences, University of Padova, via Marzolo 1, 35131 Padova, Italy
[3]Department of Physics, University of Gothenburg, Origovägen 6B, 412 96, Gothenburg, Sweden
[4] Department of Chemistry and Chemical Engineering, Chalmers University of Technology, Kemigården 4, 412 96 Göteborg, Sweden
[5] nanotech@surfaces Laboratory, Empa, Swiss Federal Laboratories for Materials Science and Technology, Überlandstrasse 129, 8600 Dübendorf, Switzerland
[6] Department of Physics, Universidad Autónoma de Madrid, Ciudad Universitaria de Cantoblanco, 28049 Madrid, Spain
[7] Department of Physics and Materials Science, University of Luxembourg, 162a avenue de la Faïencerie, L-1511 Luxembourg, Luxembourg
[8] CNR Institute of Nanoscience, via Campi 213/A, 41125 Modena, Italy
[9] Department of Physics, Umeå University, Linnaeus väg 24, 90187 Umeå, Sweden
[10] Umeå Centre for Microbial Research, Umeå University, 901 87 Umeå, Sweden
¶These authors contributed equally
*alexd@physics.gu.se; *stefano.corni@unipd.it; *nicolo.maccaferri@umu.se

## Supplementary Note 1. Sample fabrication.

### Supplementary Note 1.1 – Fabrication procedures

Quartz substrates (2 x 2 $cm^2$) were cleaned by immersing them consecutively in acetone and isopropanol in an ultrasonic bath, to be finally rinsed in DI water and dried with nitrogen. Hole-mask colloidal lithography (HCL) **[1]** nanofabrication technique was employed to deposit Al nano-ellipses. Briefly, a PMMA layer (950 PMMA A4) was spin-coated for 60 seconds at 3000 rpm. After a brief O2 plasma treatment (5 s, 250 mTorr at 50 W), an aqueous polyelectrolyte layer of PDDA solution at 0.2% was pipetted into the surface and rinsed with DI water after 1 minute. Then, an aqueous solution of polystyrene (PS) beads of 100 nm-diameter (0.2 % in volume) were pipetted and rinsed with DI water after 2 minutes. A 10 nm Cr layer is then evaporated on top of the tilted substrate, so the shadow of the PS beads generates an elliptical-hole mask. After tape-stripping of the PS beads, O2 plasma etching (5 min, 250 mTorr at 50 W) generates the holes in the PMMA, and 60 nm e-beam evaporated Al is deposited. Finally, hot acetone in an ultrasonic bath is used in the final lift-off step. The final nanoellipses have a long axis of around 140 nm and short axis of 100 nm. The spiropyran (1,3,3-Trimethylindolino-6´-nitrobenzopyrylospiran, from Tokyo Chemical Industry) is mixed with poly(styrene) standard (MW= 45730, Aldrich Chem. Co.) (PS) in a ratio spiropyran:PS of 2:0.5 wt% and dissolved in toluene (Merck). The as-prepared solution is spinned onto the Al nanoellipse sample at 3000 rpm for 60 seconds. This generates a transparent thin film with a thickness of around 50 nm, determined by profilometry (Surface profiler Tencor AS700). The samples were characterized using scanning electron microscopy (SEM) (Zeiss Supra 55VP at 30 kV, see Supplementary Figure 1) and in tapping mode AFM (Bruker Dimension 3100 SPM, see Supplementary Figure 2).

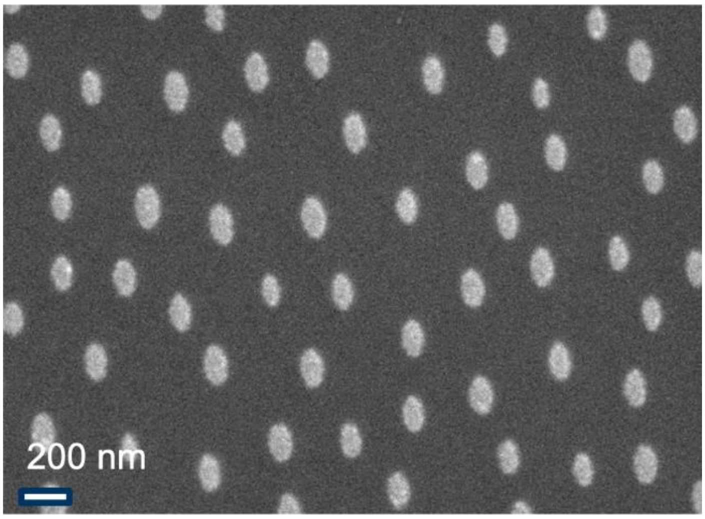


***Supplementary Figure 1:*** *SEM image of the aluminum nanoellipses prepared by hole-mask colloidal lithography.*

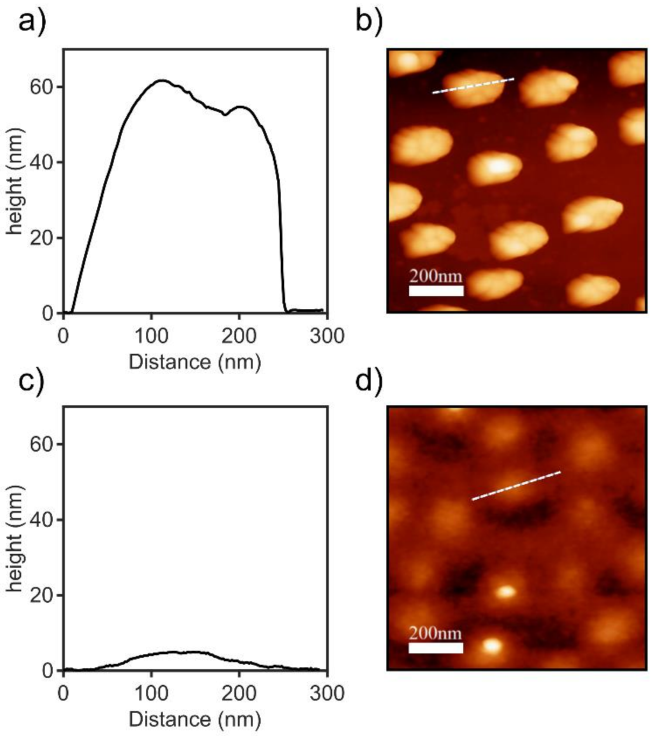


***Supplementary Figure 2:*** *AFM characterization of the samples.* ***a*** *height profile of the nanoellipse without SP layer along the dashed line indicated in (b).* ***b*** *AFM amplitude (b) measured on the sample without SP layer.* ***c,d*** *Same as (a),(b) for sample with the SP layer. Scale bar is 200 nm.*

**Supplementary Note 1.2 – Determination of the molecular density and photoconversion yield**

In order to determine the molecular density in a SP thin film, we dissolve a substrate of 1.5 x 1.5 $cm^2$ containing a film of polystyrene and SP molecules as described in Supplementary Note 1.1 (Supplementary Figure 3, black line) in 1.5 ml of acetonitrile (molar absorption coefficient for SP **[2]**: $\varepsilon$=7500 $M^{-1}$ $cm^{-1}$ at 341 nm, Absorption maximum = 0.206) in a 4 mm optical path cuvette (Supplementary Figure 3, red line). The concentration is around 68 $\mu$M, and with a film thickness of 50 nm we obtain a molecular density of around 5,5 SP molecules/$nm^3$.

Finally, we determined the photo-conversion yield of the SP isomer to the MC isomer in the thin film. Comparing the efficiency of the UV-induced SP $\rightarrow$MC isomerization in the molecular film (without ellipses) to the corresponding process in acetonitrile fluid solution using identical light flux and sample geometry, we found that the rate of ring opening in acetonitrile was a factor around 6 faster ($k_{sol}$ = 0.39 $min^{-1}$ in acetonitrile, $k_{film}$= 0.067 $min^{-1}$ in the film, 365 nm UV light at 0.9 mW/$cm^2$ at the samples). The isomerization quantum yield for this spiropyran in acetonitrile is reported to be 0.12 **[3]** implying that the corresponding number in the film is expected to be around 0.02.

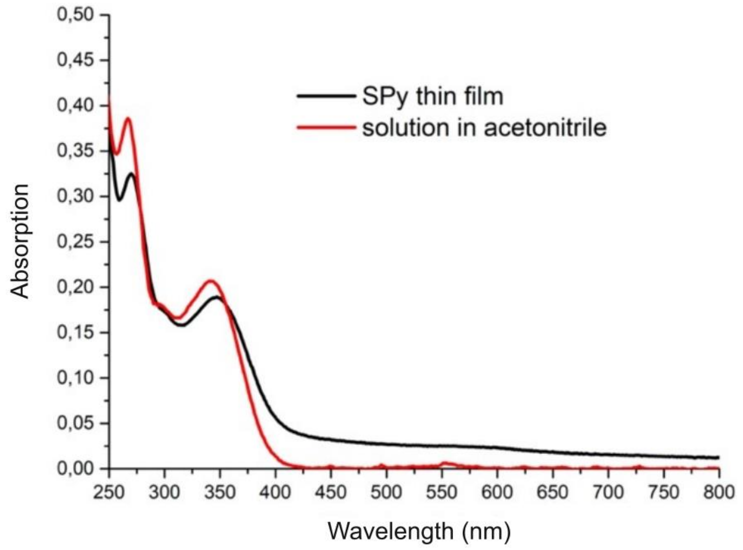


***Supplementary Figure 3:*** *Spectra associated with the determination of the molecular density in the SP thin film. Black line spectrum represents the original SP thin film and the red line the spectrum of the same film when it is dissolved in acetonitrile.*

## Supplementary Note 2. Computational part.

### Supplementary Note 2.1 – Quantum mechanical calculations of the isolated spiropyran and merocyanine molecules

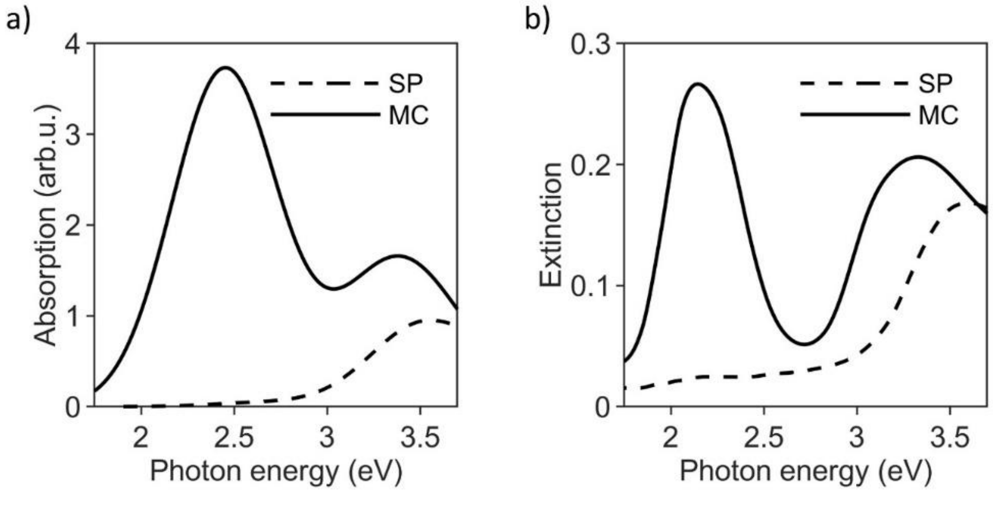


***Supplementary Figure 4:*** *a) Simulated linear absorption in Ethylbenzene at TD-DFT/B3LYP 6-31g(d,p). b) Experimental extinction spectra of the polystyrene film containing the molecules.*

The ground state structures of the two molecules were optimized at B3LYP/6-31g(d,p) level of theory using the Gaussian 16 package **[4]** in vacuum:

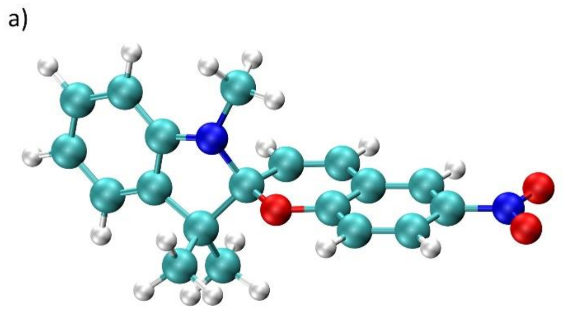


***Supplementary Figure 5**: a) Ground state optimized structure of the SP isomer. b) Ground state optimized structure of the MC isomer.*

From the two structures reported in Supplementary Figure 5, vertical excitation energies at TD-DFT/B3LYP 6-31g(d,p) for the first 8 excited states were computed, including ethylbenzene as an implicit solvent using the standard IEF-PCM **[5]** implementation of the Gaussian 16 package. The resulting simulated linear absorption (obtained with a Half-Width-Half-Height broadening of 0.333 eV for each transition) is reported above in Supplementary Figure 4, displaying a very good agreement with the experimental counterpart. In addition to the ground-state optimized structures, the $S_1$ state of the merocyanine isomer was optimized in solvent to find the closest excited-state minimum towards which the system can relax upon excitation. According to TDDFT/B3LYP 6-31g(d,p) vertical excitation energies computed at the two different nuclear geometries corresponding to ground and excited state minima (the relaxed $S_1$ structure is shown in Supplementary Figure 6 panel b), the predicted Stokes-shift is ≈ 0.4 eV thus suggesting that the red-shift of the $\Delta T/T$ pump-probe signal reported in the main text Figure 2 (≈ 0.3 eV), can indeed be assigned to stimulated emission of excited MCs relaxing towards the $S_1$ minimum.

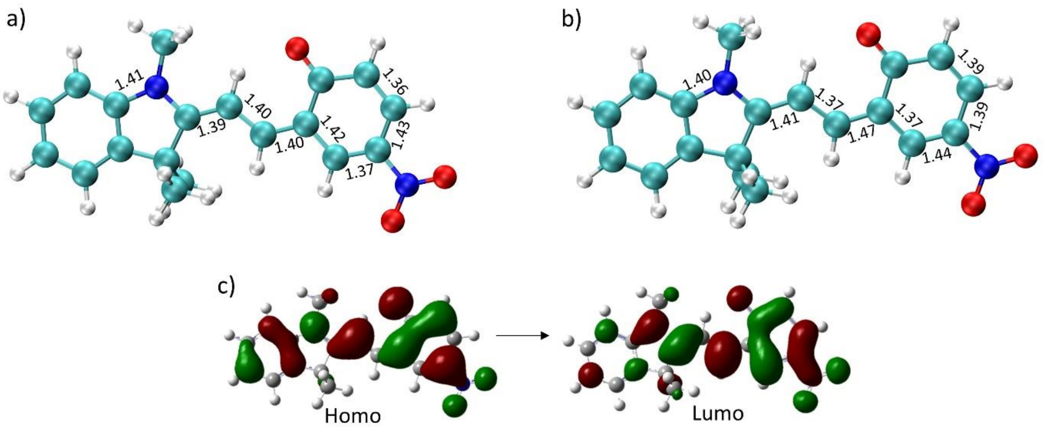


***Supplementary Figure 6:** a) Ground state optimized structure of MC b) Excited state optimized structure of MC. The bond lengths are in Å in both figures. c) $S_0 \rightarrow S_1$ bright transition of the MC (peaking at 2.45 eV in our simulations, see Supplementary Figure 4) mostly involves the HOMO → LUMO orbitals here reported.*

**Supplementary Note 2.2 – Theoretical model to simulate the optical response of the coupled system**

The nanoellipse employed in the simulations throughout this work was created using the Gmsh code **[6]** (Supplementary Figure 7).Its associated extinction spectra obtained with different polarizations of the incoming electric field are reported in Supplementary Figure 10 panel a).

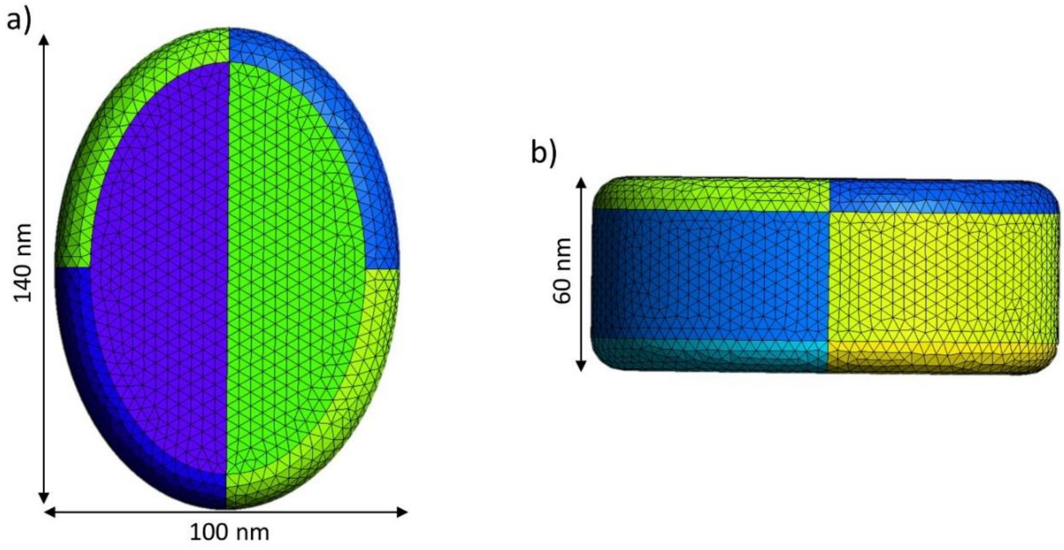


***Supplementary Figure 7:*** *Nanoellipse created with the gmsh code and employed in the simulations: front view a), side view b). The surface elements, called tessera, that are here observable result from the surface mesh discretization operated by the gmsh code and it is necessary to numerically evaluate the coupling parameters with the MC molecules, see details below.*

In order to analyze the optical properties of the hybrid light-matter states (i.e. Polaritons) mentioned in the main text, we employed the quantization scheme of the metallic response based on a single Drude-Lorentz oscillator model of the dielectric function as developed previously **[7]**. In short, starting from a classical description of the nanoparticle within the PCM-NP framework **[8]** subject to an external frequency-dependent perturbation (in the quasi-static limit), one can obtain the following expression:

$$\mathrm{q}(\omega) = \mathrm{Q}^{\mathrm{IEF}}(\omega)\mathrm{V}(\omega) \tag{1}$$

Where $\mathrm{q}(\omega)$ is a collection of polarization surface charges that account for the nanoparticle linear response under the external electrostatic potential $\mathrm{V}(\omega)$ and $\mathrm{Q}^{\mathrm{IEF}}(\omega)$ is the frequency-dependent response function, that can be recast into a diagonal form as **[9]**:

$$\mathrm{Q}^{\mathrm{IEF}}(\omega) = -\mathrm{S}^{-\frac{1}{2}}\mathrm{T}\mathrm{K}(\omega)\mathrm{T}^{\dagger}\mathrm{S}^{-\frac{1}{2}} \tag{2}$$

with

$$\mathrm{K}_{\mathrm{p}}(\omega) = \frac{2\pi + \Lambda_p}{2\pi\frac{\varepsilon(\omega)+1}{\varepsilon(\omega)-1} + \Lambda_{\mathrm{p}}} \tag{3}$$

where $\mathrm{K}(\omega)$ is the diagonal response matrix derived from eigenvalues $\Lambda_{\mathrm{p}}$ of the appropriate IEF matrix **[9]**, $\varepsilon(\omega)$ is the frequency dependent dielectric function and S is the matrix storing the electrostatic potentials between discrete points of the dielectric.
Based on this classical description and assuming a single Drude-Lorentz oscillator model for the metal dielectric function one can retrieve the following quantum response function**[7]**

$$\mathrm{Q}_{\mathrm{kj}}^{\mathrm{quant}}(\omega) = -\sum_{\mathrm{p}} \frac{\langle 0|\hat{\mathrm{q}}_{\mathrm{k}}|\mathrm{p}\rangle\langle \mathrm{p}|\hat{\mathrm{q}}_{\mathrm{j}}|0\rangle}{\omega_{\mathrm{p}} + \omega + i\Gamma/2} + \frac{\langle \mathrm{p}|\hat{\mathrm{q}}_{\mathrm{k}}|0\rangle\langle 0|\hat{\mathrm{q}}_{\mathrm{j}}|\mathrm{p}\rangle}{\omega_{\mathrm{p}} - \omega - i\Gamma/2} \tag{4}$$

where each matrix element k, j of the matrix response function $Q^{quant}(\omega)$ is evaluated on the representative points of the tesserae k and j, and $\omega_p$ is the frequency of a given plasmon mode computed as:

$$\omega_{\mathrm{p}}^2 = \omega_0^2 + \left(1 + \frac{\Lambda_{\mathrm{p}}}{2\pi}\right)\frac{\Omega_{\mathrm{p}}^2}{2} \tag{5}$$

with $\Omega_p$ being the plasma frequency of the bulk metal, and $\Gamma$ is the damping rate of the DL oscillator. It is important to notice that elements as $\langle 0|\hat{q}_k|p\rangle$ represent the quantized transition charges sitting on the k-th tessera related to the mode p, so for a given plasmon mode p a collection of quantized charges (one for each tessera) is given. These quantities, that are analogous to molecular transition densities, can be used to numerically evaluate the coupling strength between a selected plasmon mode and a given molecule, that reads:

$$\hat{\mathrm{g}}_{\mathrm{p}} = \sum_{\mathrm{j}} q_{p,j}\hat{\mathrm{V}}_{\mathrm{j}} \tag{6}$$

Where $\hat{\mathrm{V}}_{\mathrm{j}}$ is the molecular electrostatic potential operator evaluated at the position of the j-th tessera where the $q_{p,j}$ charge related to the p mode lies on.

Considering the previously mentioned theoretical background, in order to shed light on the optical response of the coupled system the aluminum nanoellipse of Supplementary Figure 7 was surrounded by many merocyanine molecules (MCs), each one described as a point-dipole and an ad-hoc Tavis-Cummings-like Hamiltonian (in atomic units, au) was set up:

$$\hat{\mathrm{H}} = \sum_{\mathrm{i}=1}^{\mathrm{N}} \overline{\omega}_{\mathrm{MC}}\hat{\sigma}_{\mathrm{i}}^{\dagger}\hat{\sigma}_{\mathrm{i}} + \overline{\omega}_{\mathrm{LA}}\hat{\mathrm{a}}_{\mathrm{LA}}^{\dagger}\hat{\mathrm{a}}_{\mathrm{LA}} + \overline{\omega}_{\mathrm{SA}}\hat{\mathrm{a}}_{\mathrm{SA}}^{\dagger}\hat{\mathrm{a}}_{\mathrm{SA}} + \sum_{\mathrm{i}=1}^{\mathrm{N}} g_{\mathrm{i,LA}}\left(\hat{\mathrm{a}}_{\mathrm{LA}}^{\dagger}\hat{\sigma}_{\mathrm{i}} + \hat{\mathrm{a}}_{\mathrm{LA}}\hat{\sigma}_{\mathrm{i}}^{\dagger}\right) + \sum_{\mathrm{i}=1}^{\mathrm{N}} g_{\mathrm{i,SA}}\left(\hat{\mathrm{a}}_{\mathrm{SA}}^{\dagger}\hat{\sigma}_{\mathrm{i}} + \hat{\mathrm{a}}_{\mathrm{SA}}\hat{\sigma}_{\mathrm{i}}^{\dagger}\right) \tag{7}$$

Where the $\hat{\sigma}_{\mathrm{i}}$ and $\hat{\sigma}_{\mathrm{i}}^{\dagger}$ are the typical molecular transfer operators and $\hat{\mathrm{a}}_{\mathrm{LA}}^{\dagger}$ ($\hat{\mathrm{a}}_{\mathrm{LA}}$) is the creation (annihilation) operator related to the long-axis mode of the nanoellipse, whereas $\hat{\mathrm{a}}_{\mathrm{SA}}^{\dagger}$ ($\hat{\mathrm{a}}_{\mathrm{SA}}$) is the corresponding operator for the short axis mode. We also note that $\overline{\omega}_{\mathrm{MC}} = \omega_{\mathrm{MC}} - i\Gamma_{\mathrm{MC}}$ , $\overline{\omega}_{\mathrm{LA}} = \omega_{\mathrm{LA}} - i\Gamma_{\mathrm{LA}}$ and $\overline{\omega}_{\mathrm{SA}} = \omega_{\mathrm{SA}} - i\Gamma_{\mathrm{SA}}$ where each imaginary component contains the corresponding decay rate of the uncoupled state. The inclusion of the decay rate as an imaginary part results in a non-Hermitian Hamiltonian, previously applied to the case of individual molecules **[10-11]** and more recently to plexcitonic systems **[12]**. The molecular decay rate is set to $\Gamma_{\mathrm{MC}} = 1.5 \times 10^{-4}\mathrm{au}$ based on the linewidth of the experimental data shown in Supplementary Figure 4, whereas $\Gamma_{\mathrm{LA}} = 0.038\mathrm{au}$ and $\Gamma_{\mathrm{SA}} = 0.053\mathrm{au}$ were chosen on the basis of the fitted DL parameters as described in Supplementary Figure 10.

In the 1-excitation-space the Hamiltonian shown above (7) reads:

$$\hat{H} = \sum_{i=1}^{N} \overline{\omega}_{MC}|G_1..E_i..G_N;0,0\rangle\langle 0,0;G_1..E_i..G_N| + \overline{\omega}_{LA}|G_1..G_N;1,0\rangle\langle 1,0;G_1..G_N| + \overline{\omega}_{SA}|G_1..G_N;0,1\rangle\langle 0,1;G_1..G_N| + \sum_{i=1}^{N} g_{i,LA}|G_1..G_N;1,0\rangle\langle 0,0;G_1..E_i..G_N| + adj. + \sum_{i=1}^{N} g_{i,SA}|G_1..G_N;0,1\rangle\langle 0,0;G_1..E_i..G_N| + adj. \tag{8}$$

Where $\mathrm{G_i}$ stands for the ground state of the i-th molecule, $\mathrm{E_i}$ means the excited state of the i-th molecule and $g_{\mathrm{i,LA}}$ ($g_{\mathrm{i,SA}}$) is the coupling strength between the i-th molecule $G_i$->$E_i$ transition and the long-axis (short-axis) plasmon mode. Note that unlike the original TC Hamiltonian, here each molecule features its own coupling strength and orientation with respect to the given plasmon modes (two simultaneously considered in this work). The values of $g_{\mathrm{i,LA}}$ ($g_{\mathrm{i,SA}}$) were calculated for each molecule by the interaction of the $G_i$->$E_i$ transition dipole with the relevant plasmonic mode charges **[7]**. Since each molecule interacts differently with the given plasmon modes, not all of them equally contribute to the polaritonic states. The Inverse Partecipation Ration (IPR) defined as $\mathrm{IPR} = \frac{1}{\sum_{\mathrm{i}=1}^{\mathrm{N}}|\mathrm{C_i}|^4}$ is a well-known quantifier for measuring exciton delocalization over many different molecules, and it can also be used in our case to have a quantitative estimate of the degree of molecular delocalization of the polaritonic states**[13]**. In the ideal limit of N identical molecules equally coupled to a given mode, the analytical solution**[17]** of the TC Hamiltonian would give a coefficient on each molecular state participating to the polaritonic wavefunction of $\frac{1}{\sqrt{2N}}$, and since in our simulation setup N is equal to 590, the corresponding IPR coefficient in the case of N molecules all equally contributing to the polaritonic states should be $\mathrm{IPR} = \frac{1}{\frac{\mathrm{N}}{4\mathrm{N}^2}} = 4\mathrm{N} = 2360$. In the LA case, the computed IPR is $\mathrm{IPR_{LA}} = 1037$, while in the SA case it is $\mathrm{IPR_{SA}} = 370$, thus clearly showing that in both cases the excitation of the collective polaritonic state is not equally shared among all molecules, since some of them are more

coupled to the modes than others. Therefore, changing the properties of a single molecule among those that are strongly contributing to the polaritonic state can provide appreciable effects in collective response.

Diagonalization of such Hamiltonian (8) gives access to the 1-excitation-space polaritonic energies (eigenvalues) and corresponding polaritonic wavefunctions (eigenvectors). Transition dipoles from the GS to each 1-excitation-space polariton (1PL), that is $\langle \mathrm{GS}|\hat{\mu}|\mathrm{1PL}\rangle = \boldsymbol{\mu}_{\mathbf{1PL}}$ , were calculated by linear combination of molecular terms obtained by the TDDFT calculations of the previous section, such as $\langle \mathrm{GS}|\hat{\mu}|\mathrm{E_1..G_N};0,0\rangle = \boldsymbol{\mu}_{\mathbf{1}}$ , and plasmonic terms, like $\langle \mathrm{GS}|\hat{\mu}|\mathrm{G_1..G_N};1,0\rangle = \boldsymbol{\mu}_{\mathbf{LA}}$ and $\langle \mathrm{GS}|\hat{\mu}|\mathrm{G_1..G_N};0,1\rangle = \boldsymbol{\mu}_{\mathbf{SA}}$ . Such plasmonic transition dipoles are evaluated as $\sum_{\mathrm{j}} q_{p,j}\mathbf{r_j}$ ,where $\mathbf{r_j}$ is the position of the center of each tessera where the quantized surface charge $q_{p,j}$ lies on , for a given mode p.

The decay rates (linewidths) $\Gamma_{\mathrm{1PL}}$ associated to each polaritonic state are directly obtained from the imaginary component of the eigenvalues of the Hamiltonian (8).

Once these quantities are computed, the linear response expression of the polarizability of the entire system (molecule+nanostructure) as a sum over the polaritonic states reads:

$$\alpha_{\mathrm{ij}}(\omega) = \sum_{\mathrm{1PL}} \frac{\langle \mathrm{GS}|\hat{\mu}_{\mathrm{i}}|\mathrm{1PL}\rangle\langle \mathrm{1PL}|\hat{\mu}_{\mathrm{j}}|\mathrm{GS}\rangle}{\omega_{\mathrm{1PL}} + \omega + i\Gamma_{\mathrm{1PL}}/2} + \frac{\langle \mathrm{GS}|\hat{\mu}_{\mathrm{i}}|\mathrm{1PL}\rangle\langle \mathrm{1PL}|\hat{\mu}_{\mathrm{j}}|\mathrm{GS}\rangle}{\omega_{\mathrm{1PL}} - \omega - i\Gamma_{\mathrm{1PL}}/2} \tag{9}$$

with $\langle \mathrm{GS}|\hat{\mu}_{\mathrm{i}}|\mathrm{1PL}\rangle = \boldsymbol{\mu}_{\mathbf{1PL,i}}$ being the i-th component of the transition dipole to a given polaritonic state 1PL with energy $\omega_{\mathrm{1PL}}$ and damping rate $\Gamma_{\mathrm{1PL}}$. Such quantity (9) is used to evaluate the absorption cross section (reported in panels e-f, Figure 1 main text) through **[14]**:

$$\sigma_{\mathrm{ii}}(\omega) = \frac{4\pi\omega}{c}\mathrm{Im}\{\alpha_{\mathrm{ii}}(\omega)\} \tag{10}$$

where ii is either xx or yy in our simulations for the LA or SA case, respectively. This is because the experiments are done with polarized light pulses, so only some components of the polarizability tensor become relevant.

On a side note, we observe that the simulated spectra in the LA case (Figure 1e, main text) features a Rabi splitting that well reproduce the experimental one (Figure 1c) even though the simulated peaks appear to be broader. Indeed, by fitting the bare plasmonic experimental extinction spectrum with a Lorentzian function (see Supplementary Figure 11) we obtain a linewidth $\Gamma$ of ≈ 0.022 au. ($\tau$ ≈ 1.1 fs) that is slightly smaller than the corresponding simulated value (Supplementary Figure 10). On these grounds, to quantitatively assess which coupling regime we are dealing with in the LA case (which is the case corresponding to zero detuning between plasmon energy and molecules energies), we resort to the well-known ratio**[15]** $\frac{2\Omega_{\mathrm{rabi}}}{\Gamma_1+\Gamma_2}$, with $\Omega_{\mathrm{rabi}}$ being the energy separation of the polaritonic states and $\Gamma_1, \Gamma_2$ being the decay rates of the uncoupled system.

Considering that $\Gamma_1 = \Gamma_{\mathrm{LA}} = 0.022$ au , $\Gamma_2 = \Gamma_{\mathrm{MC}} = 1.5 \times 10^{-4}$au ($\tau_2$ ≈ 150 fs) and that the computed rabi splitting value is ≈ 410 meV (≈ 0.015 au) , we obtain $\frac{2\Omega_{\mathrm{rabi}}}{\Gamma_{\mathrm{LA}}+\Gamma_{\mathrm{MC}}}$ ≈ 1.4, thus corroborating that we are

at least undoubtedly at the onset[16] of a strong coupling regime, and proper polaritonic states are formed in the LA case.

In order to simulate the pump-probe transient response shown in Figure 3 main text, ground state bleaching (GSB), stimulated emission (SE) and excited state absorption (ESA) terms of eq.1 (Numerical calculations section of main text) have to be computed. The GSB signal comes from eqs. 9-10, whereas the ESA term can be computed through the 2-excitation-space states (2PL), thus the following Hamiltonian has to be diagonalized:

$$\begin{aligned}\hat{\mathrm{H}} = &\sum_{i=1}^{N}\sum_{j>i} 2\bar{\omega}_{\mathrm{MC}}\left|\mathrm{E_iE_j..G_N};0,0\right\rangle\left\langle 0,0;\mathrm{E_iE_j..G_N}\right| + \sum_{i=1}^{N}(\bar{\omega}_{\mathrm{LA}}+\bar{\omega}_{\mathrm{MC}})\left|\mathrm{E_i..G_N};1,0\right\rangle\left\langle 1,0;\mathrm{E_i..G_N}\right| \\ &+ 2\bar{\omega}_{\mathrm{LA}}\left|\mathrm{G_1..G_N};2,0\right\rangle\left\langle 2,0;\mathrm{G_1..G_N}\right| + \sum_{i=1}^{N}\sum_{j>i} g_{\mathrm{j,LA}}\left|\mathrm{E_iE_j..G_N};0,0\right\rangle\left\langle 1,0;\mathrm{E_iG_j..G_N}\right| \\ &+ \mathrm{adj.} + \sum_{i=1}^{N}\sqrt{2}g_{\mathrm{i,LA}}\left|\mathrm{E_i..G_N};1,0\right\rangle\left\langle 2,0;\mathrm{G_i..G_N}\right| \\ &+ \mathrm{adj.} + \text{corresponding terms of SA mode}\end{aligned} \quad (11)$$

We note that considering the 2-excitation-space polaritons, in addition to the more canonical 1-excitation-space states, was recently suggested to be a theoretically robust way for interpreting transient pump-probe data of molecules in QED cavities by A. DelPo et al. [17]. We also remark that eigenenergies and eigenstates of our Hamiltonian (11) exactly match the analytical results reported in their work in the limit of identical molecules and couplings with all transition dipoles oriented along the direction of the cavity mode.

As mentioned in the main text, we assume that the pump-induced excitation of the polaritonic state (see Figures 3-4, main text) leads to the population of one localized molecular state $|\mathrm{MC}^*\rangle$ upon plasmon dephasing, from which SE to the GS and ESA to the 2-excitation-space polaritons transiently take place. The SE spectrum is simulated using (9) and (10), based on the wavefunction of the emitting state $|\mathrm{MC}^*\rangle$ obtained through diagonalization of the 1-excitation Hamiltonian (8) with one red-shifted MC. Indeed, the formation of this localized state $|\mathrm{MC}^*\rangle$ comes from diagonalization of the Hamiltonian (8) once one of the diagonal elements $\bar{\omega}_{\mathrm{MC}}$ corresponding to one MC molecule that was originally contributing most to the collective state gets red-shifted as $\bar{\omega}_{\mathrm{MC}^*} = \bar{\omega}_{\mathrm{MC}} - \delta$ (with $\delta$ being the frequency shift because of vibrational relaxation, see Numerical calculations section, main text) and it can be easily understood in the framework of perturbation theory. In the LA case ,where $\omega_{\mathrm{MC}} = \omega_{\mathrm{LA}}$, the largest computed single-molecule coupling with the LA mode is ≈ 18 meV (after scaling the actual value by a factor of 15, see Supplementary Figures 8-9 below and related discussion), so as $\delta$ increases (for instance it is already ≈ 50 meV at 50 fs after the pump excitation, see 1-to-1 mapping in Numerical calculation section of main text) we approach a perturbative regime where $\frac{g}{\delta} \ll 1$ , therefore by diagonalizing the Hamiltonian (8) with such δ values we basically get one state corresponding to the red-shifted molecule, and collective polaritonic states of the N-1 remaining molecules, along with their associated dark states. In the SA case this regime is reached even more quickly as before applying any frequency shift there is already a zero-order frequency detuning between $\omega_{\mathrm{MC}}$ and $\omega_{\mathrm{SA}}$ of ≈ 300 meV.

On the other hand, the ESA term can be obtained through equation (10), having:

$$\alpha_{ij}(\omega) = \sum_{2PL} \frac{\langle MC^*|\hat{\mu}_i|2PL\rangle\langle 2PL|\hat{\mu}_j|MC^*\rangle}{(\omega_{2PL} - \omega_{MC^*}) + \omega + i|\Gamma_{2PL} - \Gamma_{MC^*}|/2} + \frac{\langle MC^*|\hat{\mu}_i|2PL\rangle\langle 2PL|\hat{\mu}_j|MC^*\rangle}{(\omega_{2PL} - \omega_{MC^*}) - \omega - i|\Gamma_{2PL} - \Gamma_{MC^*}|/2} \tag{12}$$

Where the 2-excitation-space polaritons are in this case obtained through diagonalization of (11) with all the diagonal elements corresponding to the red-shifted MC molecule modified accordingly to account for the frequency shift (similarly to the frequency shift shown above for the 1-excitation Hamiltonian, but this time applied to all diagonal elements of (11) where that molecule appears). Since Figure 3 (main text) presents data at different time delays, which corresponds in our picture to different frequency shifts because of vibrational relaxation, the diagonalization of (8) and (11) with some red-shifted diagonal elements have been performed multiple times, each time for a different frequency shift value corresponding to a given time delay, following the 1-to-1 mapping mentioned in the Numerical calculations section of main text.

We remark that the N-1 simplified picture that we also make use of in the main text for explaining the results follows directly from this complete model once the shift is such that $\frac{g}{\bar{\omega}_{LA/SA} - \bar{\omega}_{MC^*}} \ll 1$. Under this regime, diagonalization of (8) leads to the localized molecular state $|MC^*\rangle$ on which the initial collective excitation is collapsed, and from this state the bright states that can be populated upon probe absorption (ESA contribution) which are obtained by diagonalizing (11) with corresponding shifted diagonal elements can be mostly seen as polaritonic states of the N-1 remaining molecules.

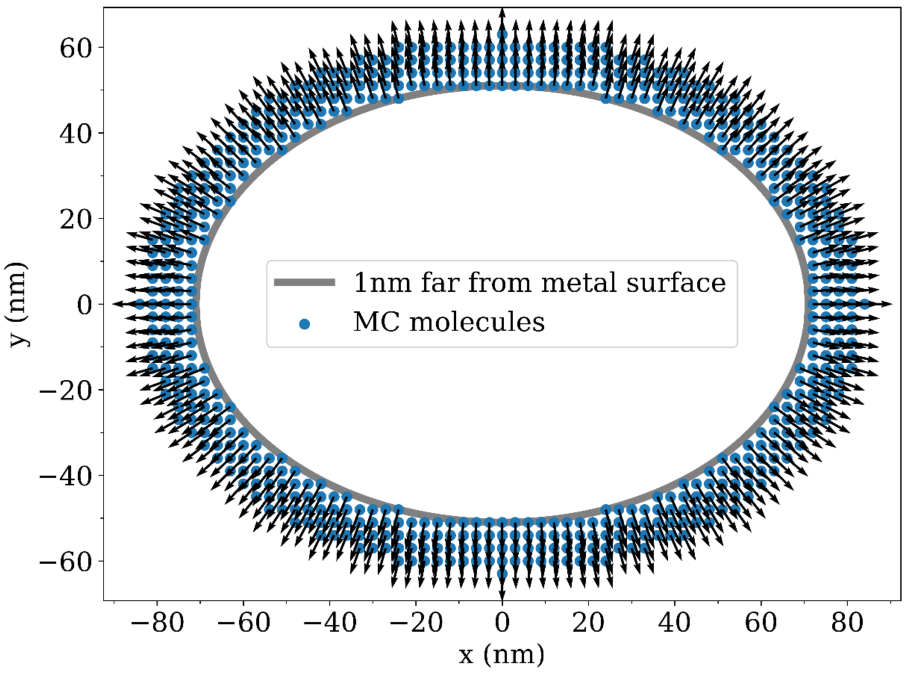


***Supplementary Figure 8:*** *2D slice of the elliptical grid employed in the simulations. Each blue dot represents a MC molecule described as a point-dipole oriented perpendicular to the metal surface. The nearest neighbor distance was set to 3 nm, which corresponds to the sample concentration of 2% wt (≈ 1 molecule per 26 nm$^3$). Data provided in the main text was obtained with such setup on a 2D layer of molecules only, scaling the coupling values accordingly to match with the experimental linear absorption of the coupled system. More detailed information regarding the computed coupling strength with the full 3D grid are reported in Supplementary Figure 9.*

As described in Supplementary Figure 8, the data reported in the main text was obtained considering a single 2D grid layer of molecules located at half height of the nanoellipse to save computational time, and the corresponding calculated coupling values have been increased until a good matching with the experimental linear absorption was found (see Figure 1 main text). (We recall the relation between the Rabi splitting energy $\Omega_{\mathrm{R}}$ and the number of molecules $N_{\mathrm{res}}$ resonantly coupled to the mode in the simplified case of equal coupling for all the molecules, i.e., $\Omega_{\mathrm{R}} \propto \sqrt{N_{\mathrm{res}}}$ ).

Despite this computationally convenient choice, calculations with full 3D grid of MCs surrounding the nanoellipse were also performed in order to assess whether the computed couplings of the real 3D system were large enough to correctly reproduce the experimental data (without any ad hoc increase). As shown in Supplementary Figure 9 panel a), two distinct grid steps have been tested, 3.0nm (corresponding to 1 molecule per 27 $nm^3$) and 2.0 nm (corresponding to 1 molecule per 8 $nm^3$) respectively, and it turned out that in the tightest 3D grid case (2 nm step size, green curve), by employing a multiplying factor of 2 for the computed couplings, the corresponding Rabi splitting is slightly larger than the experimental one, which perfectly matches with that predicted assuming 1 single 2D layer with step size of 3nm and couplings scaled by a factor of 15 (blue curve). These numerical tests point out that in the realistic limit of a full 3D grid having an intermediate step size between 2 and 3 nm the calculated couplings would reproduce the experimental data, thus corroborating the theoretical model.

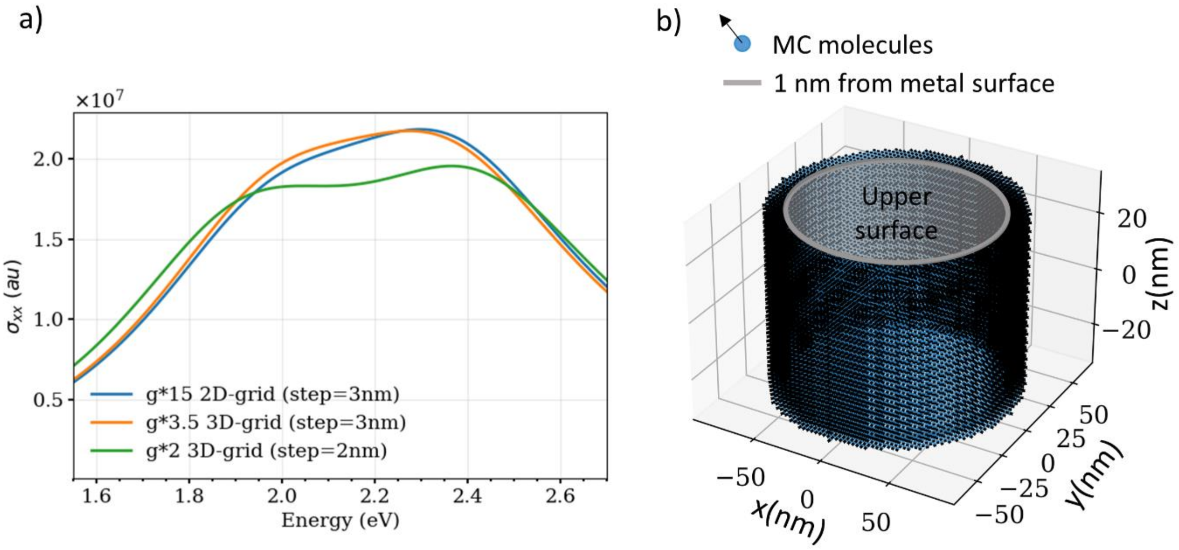


***Supplementary Figure 9:*** *a) Simulated linear absorption of the polaritonic system (LA case) considering different grids and scaling factors for the computed couplings: 2D grid only (step size 3 nm between nearest neighbors and couplings scaled by a factor of 15) (blue line), 3D grid (step size 3nm and couplings scaled by a factor of 3.5) (orange line) and 3D grid (step size 2nm and couplings scaled by a factor of 2). b) Scheme of the 3D grid employed to obtain the orange line panel a.*

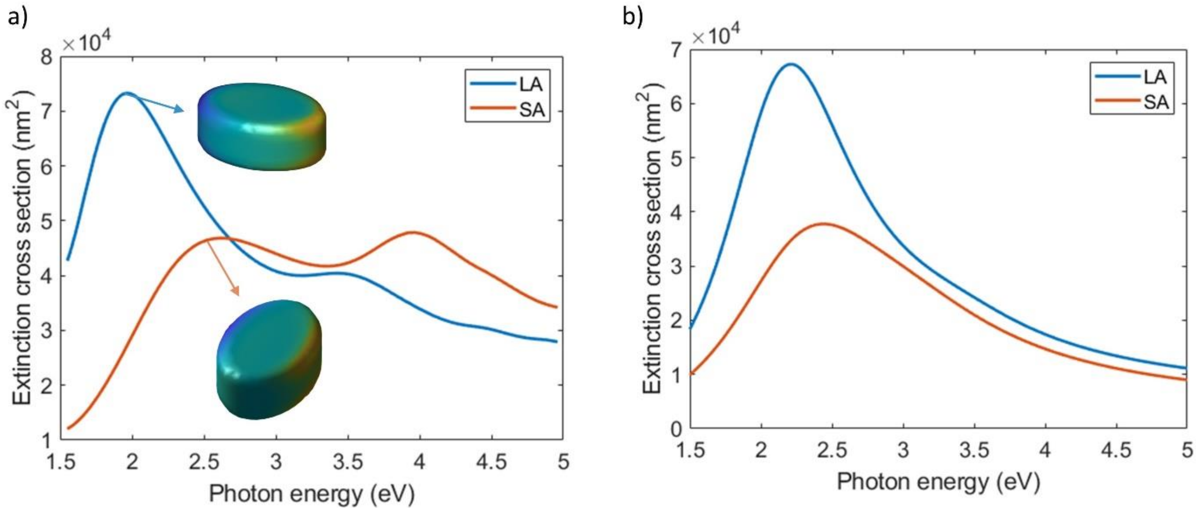


***Supplementary Figure 10:*** *a) Simulated extinction spectra of the model nanoellipse computed by solving the full Maxwell equations through the MNPBEM code* ***[18]*** *for two distinct electric field polarizations of the incoming wave: long-axis direction (blue line) and short-axis direction (orange line). The aluminum dielectric function employed in the simulations is that of McPeak et al.* ***[19]*** *and the environment refractive index was set to 1.5. The two small insets display the surface charge distribution associated with the corresponding main peaks (dipolar plasmon resonances of interest); the yellow color represents positive charges, whereas blue represents negative ones. The two peak energies are very close to the experimental ones (2.15 and 2.45 eV after Spy deposition, Figure 1 panels c-d main text). b) Simulated quasistatic extinction spectra of the same nanoellipse by adopting a single Drude-Lorentz oscillator model for the dielectric function and tuning the corresponding parameters to reproduce the principal features of the two main dipolar resonances shown in panel a). The plasma frequency was set to 0.025 au (eq. 5) and the following decay rates* $\Gamma_{LA}$*=0.038 au and* $\Gamma_{SA}$*=0.053 au for the long-axis (LA) and short-axis (SA) plasmons were employed.*

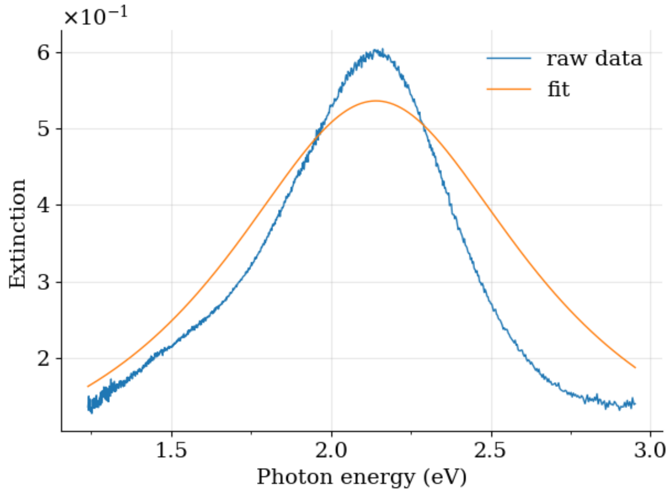


***Supplementary Figure 11:*** *Experimental plasmonic extinction spectrum in the LA case (blue curve) and corresponding fitted curve (orange) obtained with a Lorentzian function:* $\frac{\Gamma}{\pi*((\omega-\omega_0)^2+\Gamma^2)}$*. The fitting procedure yielded* $\Gamma$*≈0.022 au (≈600 meV) that is somewhat smaller than the value obtained from simulations (Supplementary Figure 10).*

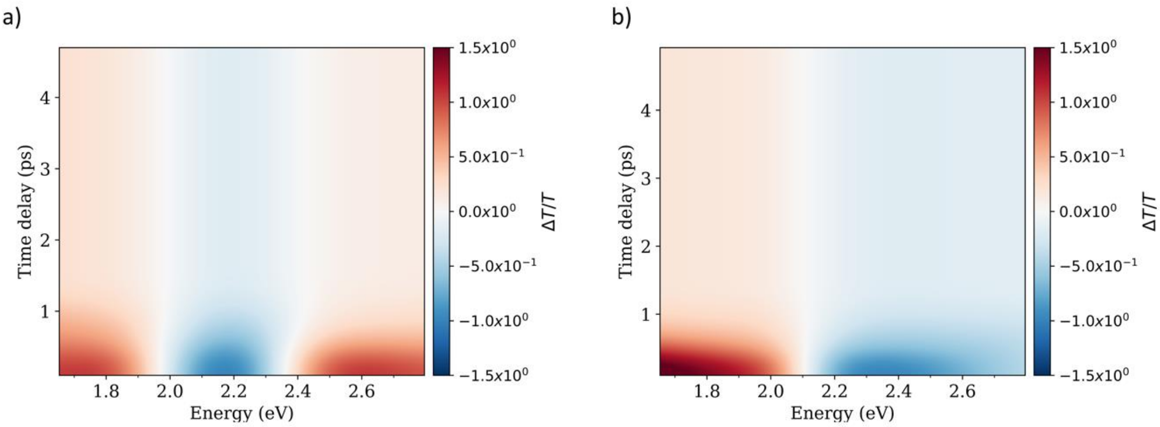


***Supplementary Figure 12:*** *Pump-probe simulated 2D maps in the LA (a) and SA (b) case according to the procedure described in the Numerical calculations part of the Methods section, main text. Since the experimental signal shown in Figure 3 a-d (main text) presents a constant magnitude after ≈1 ps that is roughly 1/5 of the intensity at early times, the same constant feature has been applied here to recover a similar spectral trend at longer times (> 1ps).*

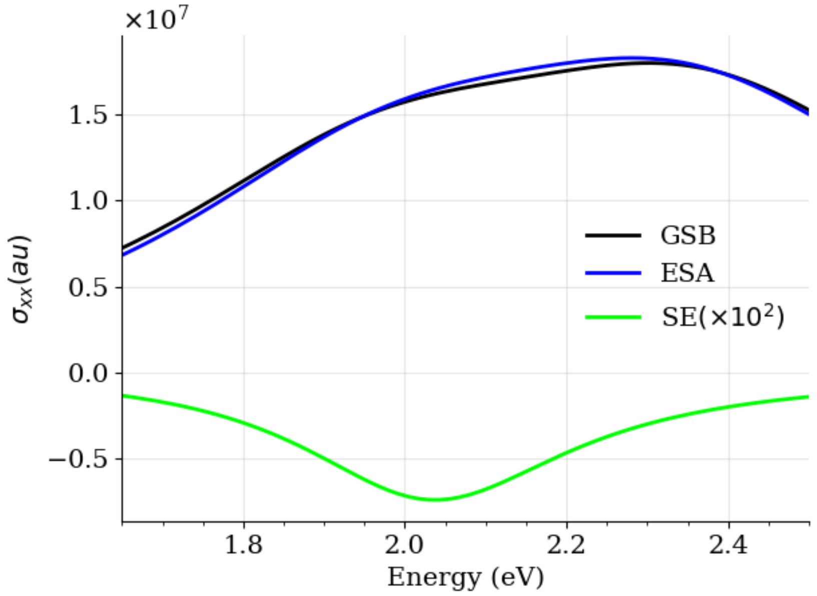


***Supplementary Figure 13:*** *Explicit GSB, ESA and SE contributions obtained through the theoretical model at 200 fs from the pump excitation. The time delay corresponds to a merocyanine frequency shift of 125 meV (see 1-to-1 mapping between molecular energy shifts and time-delay in the Numerical calculations section of main text). The three contributions are combined according to eq.1 (main text) to get the transient data reported in Figure 3c (main text). Note that GSB and ESA appear with opposite signs in that equation. The SE contribution is originating from a single molecule state (see Figure 4, main text), indeed it is considerably smaller than GSB or ESA contributions which are the predominant terms (the SE signal has been scaled by $10^2$ in the plot to ease visual inspection).*

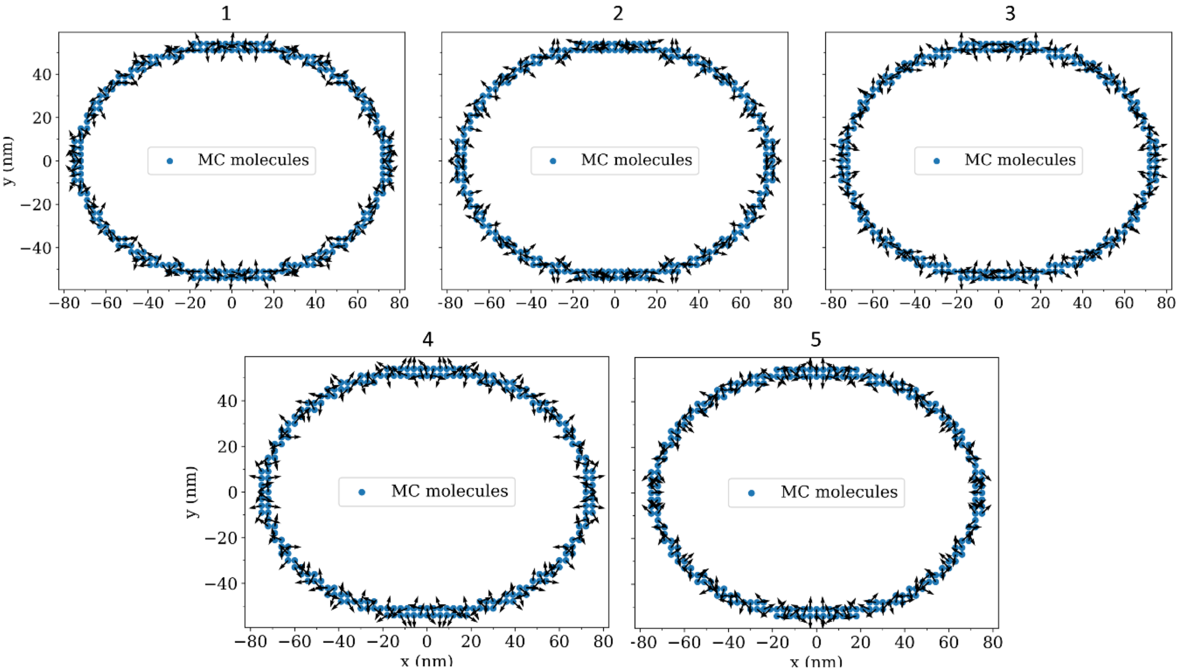


***Supplementary Figure 14:*** *Different (1-5) randomly oriented merocyanines (here represented as point-dipoles) used to obtain the results reported in Supplementary Figure 12 to test the robustness of the theoretical simulations against different alignments of the dipoles with respect to the nanoantenna.*

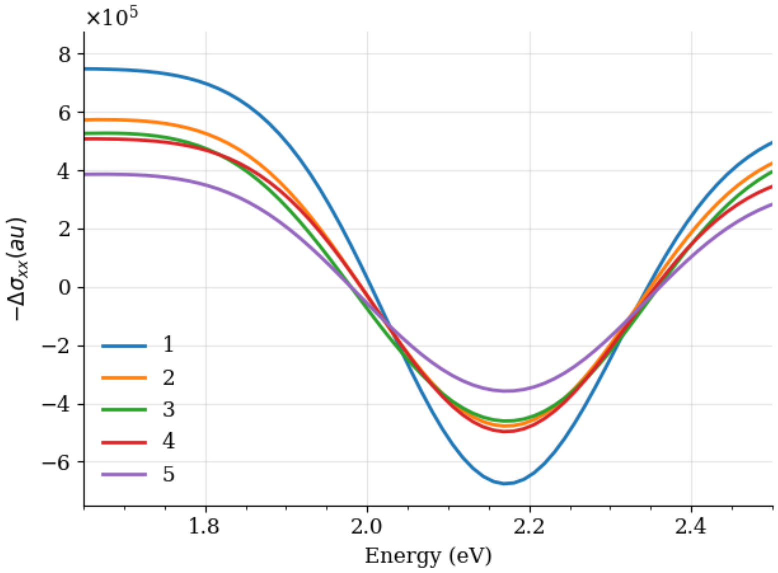


***Supplementary Figure 15:*** *Simulated transient response in the LA case for each (1-5) set of randomly distributed dipoles of Supplementary Figure 14 considering a frequency shift of 100 meV for one MC molecule. Although some differences are visible, the overall qualitative signal trend always match with the experimental one (Figure 3 a-c, main text). In particular, we note that no spectral shift is observed for the different configurations, thus proving that dipoles' orientation does not affect the interpretation of the ultrafast dynamics observed.*

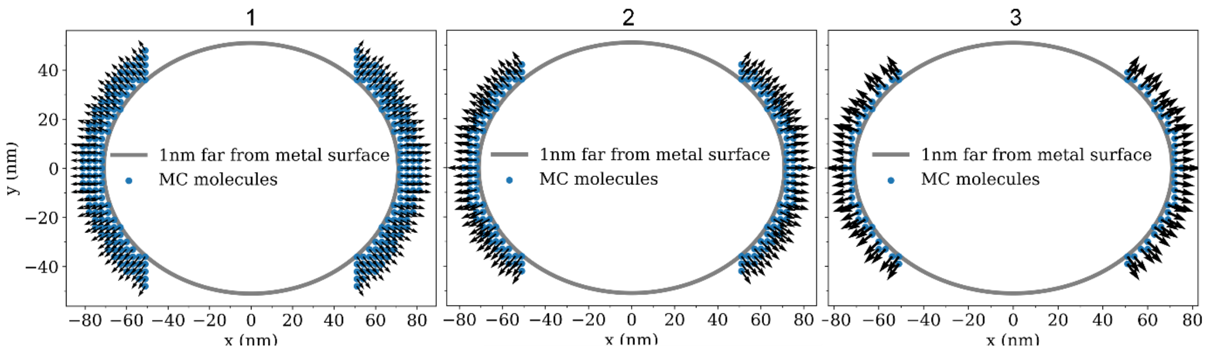


***Supplementary Figure 16:*** *Different thicknesses of the molecular layer around the nanoantenna (1-3). From 1 to 3 the thickness decreases and so does the number of emitters (merocyanines, here represented as point dipoles) coupled to the LA mode.*
*The corresponding simulated linear and transient optical signals are reported in Supplementary Figure 17.*

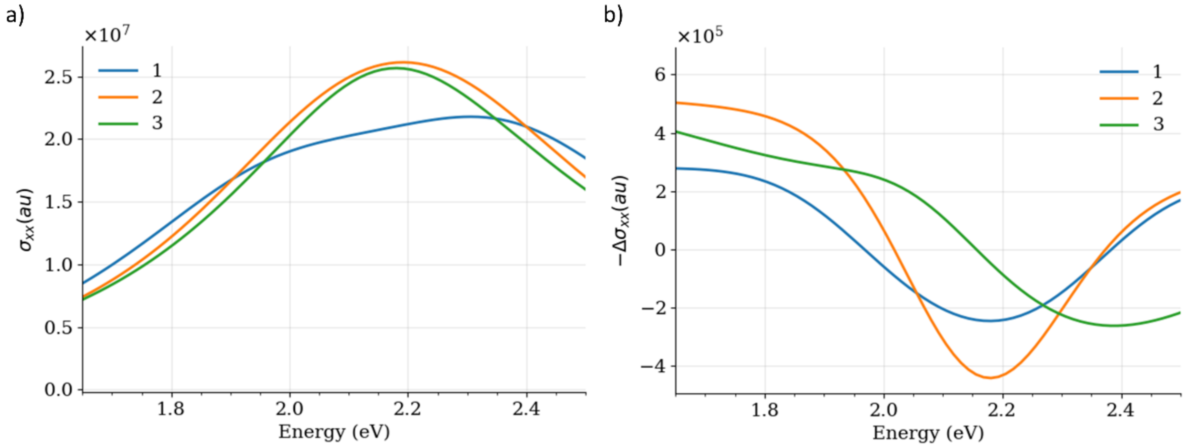


***Supplementary Figure 17:*** *Simulated linear(a) and transient(b) spectra of the corresponding setups of Supplementary Figure 16. The transient data of panel b is obtained considering a frequency shift of 125 meV for a single MC molecule, which would correspond to the signal probed at 200 fs (see Numerical calculations section of main text for the energy-to-time delay mapping). All simulations have been performed by scaling the molecule-nanoantenna couplings so that setup n.1 yields a linear absorption matching the one of Figure 1c (main text) that was obtained considering a thicker layer of molecules (Supplementary Figure 8). Notably, as the molecular layer thickness (and so the number of emitters) decreases, the simulated transient signal (panel b) seems to approach a spectral line shape (green curve) that resembles what we get for the SA case (Figure 3f main text), which is the weakly-coupled system.*

**Supplementary Note 2.3 – Theoretical discussion on the experimental results of Figure 5 (main text)**

The data shown in Figure 5 (main text) illustrate the presence of an accelerated molecular decay both in the weakly (Figure 5b) and strongly coupled case (Figure 5c) compared to isolated molecules (Figure 5a). Interestingly, by following the wavefunction composition of the localized molecular state in the LA case during the vibrational dynamics we noticed that a residual coupling with the plasmon is still present. Notably, this coupling slightly increases when the localized molecular state approaches the energy of the lower polariton state of the remaining N-1 molecules. On this ground, we can reasonably relate the observed accelerated decay of the localized excitation to a plasmonic

effect, leading to the sub-ps decay that is shown in Figure 5b-c. Indeed, this residual plasmonic component leads to a fast decay that can be estimated to be $\Gamma_{\mathrm{MC}^*} \approx \left|C_{\mathrm{MC}^*-\mathrm{p}}\right|^2 * \Gamma_{\mathrm{LA}}$ (where $C_{\mathrm{MC}^*-\mathrm{p}}$ is the coefficient of the localized state wavefunction on the LA plasmonic state, whose squared modulus is ≈ 3.5*10^-3^, and $\Gamma_{\mathrm{LA}} = 0.038\,\mathrm{au.}\,(\tau \approx 0.6\,\mathrm{fs})$ is the LA plasmon decay rate, Supplementary Figure 10) which corresponds to a lifetime of ≈ 0.2 ps.

A similar reasoning for the short axis case (SA) leads to a comparable value.

Additionally, we can (although just theoretically) decompose such enhanced decay in a radiative and a non-radiative contribution. The radiative contribution can be estimated as**[20]**:

$$\Gamma_{\mathrm{rad}}^{\mathrm{LA}} = \frac{4}{3}\frac{\omega^3}{c^3}|\boldsymbol{\mu}_{\mathbf{tot}}|^2 \tag{13}$$

With $\boldsymbol{\mu}_{\mathbf{tot}}$ being the total dipole of the localized state calculated as described in section 2.2, which then presents a plasmonic contribution as mentioned above, and hence intrinsically accounts for the Purcell-enhanced radiative emission. In both the LA and SA cases we obtain that $\Gamma_{\mathrm{rad}}^{\mathrm{LA/SA}}$ is almost an order of magnitude smaller than the corresponding value of $\Gamma_{\mathrm{MC}^*}$ , pointing to the enhanced non-radiative decay as prevailing.

## Supplementary Note 3. Time-resolved experiments.

### Supplementary Note 3.1 – Nonlinear optical response of Spiropyran and influence of photoisomerisation in pump-probe experiments

To study the transient transmission of merocyanine molecules, as shown in the main manuscript, the molecular film is illuminated with UV light that photoswitches SP molecules to their MC isomer. The pump-probe measurements are then performed on the switched sample. Via continuous spectrum acquisition, we can track both the nonlinear optical response $\Delta T/T$ and the linear transmission $T$ simultaneously during the pump-probe measurement. The linear Transmission $T$ at 2.15 eV during one such measurement on the merocyanine thin film is shown in Supplementary Figure 18 a). After switching on the UV light source at time $t = 0$, $T$ decreases rapidly due to activation of the MC $\pi - \pi^*$ transition. Subsequently, $T$ increases again due to back-photoswitching of MC to the SP form induced by irradiation with the optical pump and probe pulses. Hence, this should lead to less MC molecules contributing to the pump-probe signal over time. We can track the magnitude of the pump-probe signal by repetitively performing the same pump-probe scan while monitoring the transient transmission $\Delta T/T$. More in detail, the 2D map shown in Figure 2 a) of the main manuscript is averaged in energy from 1.75 eV to 2.25 and then averaged in time from 0.5 ps to 5 ps, providing a measure of the signal magnitude of the individual pump-probe scan. The data for repetitive measurements is shown in Supplementary Figure 18 b). The source of the observed decrease is a decrease in the number of MC molecules due to visible-light-induced back-photoswitching to the SP form. Hence, we observe the same time constant of the decay as shown in a). Indeed, the SP molecules do not contribute to the pump-probe signal, as shown in Supplementary Figure 18 c), where a pump-probe measurement is performed on the SP molecular film without prior UV illumination. Note that a small change of Δ T/T can be observed at the temporal overlap of pump and probe pulses. Such artefact can be caused by coherent interaction of pump and probe pulses mediated by higher-order susceptibilities of the molecular film**[21]**. However, such coherent artefacts are localized at t=0 and

thus do not contribute to the pump-probe signal at t>0. This allows a mapping of the pump-probe signal strength to the number of molecules that are still in their MC form. Thus, in order to correct for the decreasing number of molecules, the pump-probe data can be renormalized by the curve shown in a).

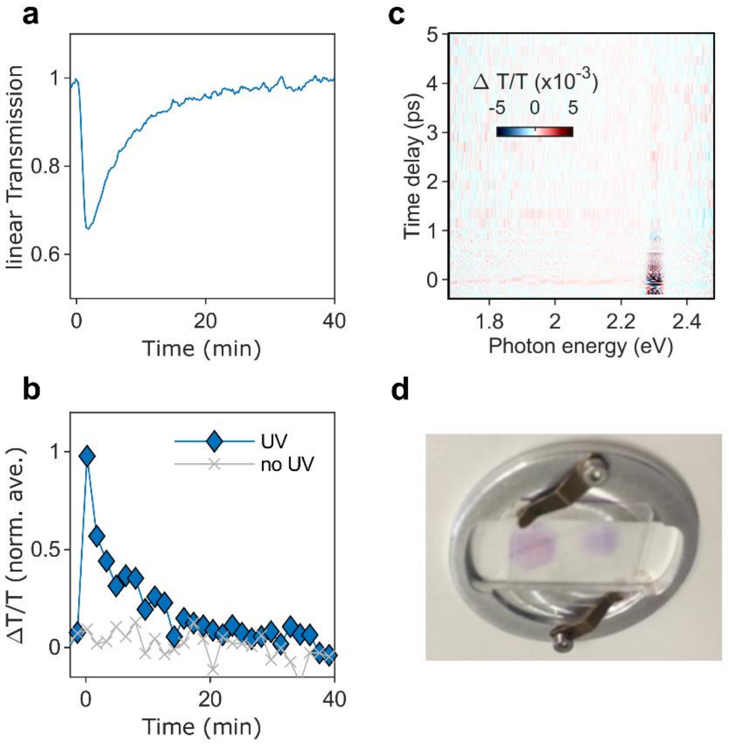


***Supplementary Figure 18:*** *a) Linear transmission of the molecular film during pump-probe measurements. The UV light source is switched on at t=0 min. Illumination with visible light pulses causes MC molecules to switch back to the SP form. b) Energy(1.75-2-25 eV)- and time(0.5-1 ps)-averaged* $\Delta T/T$ *for repetitive pump-probe scans (blue diamonds) on the UV illuminated sample. Grey stars is data recorded on the film without UV illumination. c) 2D pump-probe spectrum acquired on the SP molecules without UV illumination. d) Photograph of the sample 2 days after performing ~100 pump-probe scans. Irradiated spots appear with a purplish color, indicative of permanent damage of the sample.*

Finally, after performing many measurements on the same spot on the sample, a permanent change of color can be observed (Supplementary Figure 18 d), related to photodegradation of the molecular film. Such photodecomposition, i.e. irreversible side reactions that lead to unwanted byproducts, was also found in previous works **[22]**. Usually, up to $\sim 10^8$pump pulses (~1h of measurements) can be applied to one spot before permanent damage. To obtain measurements of the pristine sample, the sample can then be moved, such that a new spot is irradiated.

**Supplementary Note 3.2 – Determination of vibrational relaxation time scale**

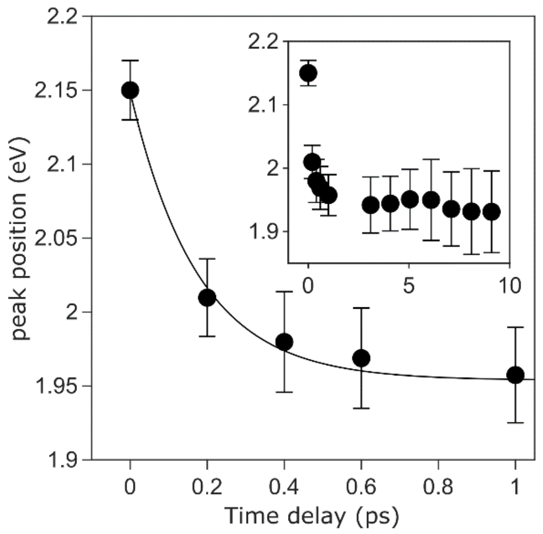


***Supplementary Figure 19:*** *Peak positions of $\Delta T/T$ obtained via fitting of energy-resolved cuts of the 2D pump-probe data shown in Figure 2 a) of the main manuscript with a Gaussian spectral profile. Error bars represent 95% confidence bounds and are obtained from the Gaussian fit. Inset is a zoom-out of the data. A red-shift due to vibrational relaxation on the MC excited state is clearly reflected by the data. Solid line shows an exponential fit to the data.*

In order to determine the time scale of the molecular vibration on the MC excited state, energy-resolved cuts shown in Figure 2 of the main manuscript are fitted with a Gaussian profile, allowing to obtain the peak position of $\Delta T/T$ for each time delay (see Supplementary Figure 19). Clearly, the red-shift that is observed in the 2D map shown in Figure 2 a) of the main manuscript is reflected by the fitted peak positions. Solid line shows an exponential fit yielding a time constant of $(200 \pm 100)$ fs.

**Supplementary Note 3.3 – Ultrafast response of aluminum ellipse antennas**

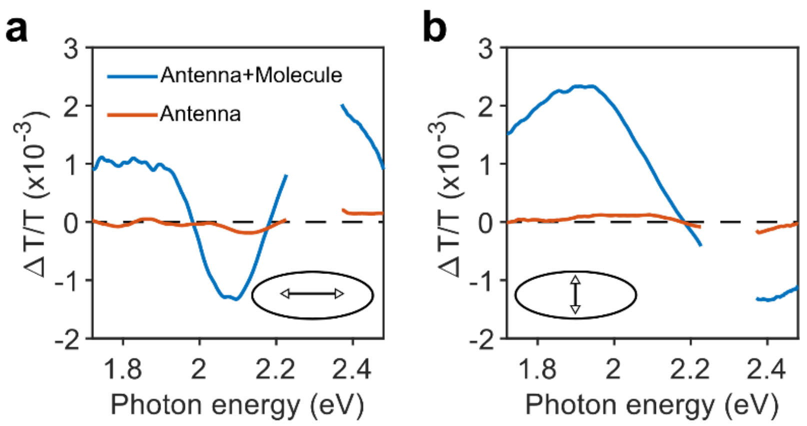


***Supplementary Figure 20:*** *Spectrally resolved $\Delta T/T$ at pump-probe time delay of 150 fs for excitation along the long (a) and short (b) axis of the antenna. Blue curves are recorded after UV switching of SP to MC and orange curves are recorded without UV switching.*

In order to characterize individual elements composing the polaritonic system, we further studied the dynamic response of the aluminum ellipse antennas to the optical pump. Before coating the antenna array with the molecular film, the plasmonic resonances lie outside of the spectral region that is probed in our experiments, due to static change in the environmental refractive index. Hence, to mitigate this issue, we performed pump-probe experiments on the antenna array without UV switching the molecular film to the MC form. Since there is no observable dynamics of the molecules in their SP form (compare Supplementary Figure 18 panel b and c), this allows to retrieve the antennae's response in their realistic environment. Results before (orange) and after UV switching (blue) are shown in Supplementary Figure 20 for long and short axis excitation. A small differential signal can be observed for the antenna response (orange), most likely due to local heating of the metal leading to a spectral shift of the plasmonic resonances. However, this contribution of the antenna to the transient signal is at least one order of magnitude reduced compared to the response of the hybrid system, confirming that the effect observed in our experiments really stems from the interaction of the plasmonic and molecular systems.

**Supplementary Note 3.4 – Fitting procedure of pump-probe time traces**

To obtain fitted curves presented in Figure 5 of the main manuscript, single-,

$$M(t) = \frac{1}{2}\left[erf\left(\frac{t-t_0}{\sigma}\right)+1\right]\cdot\left[Aexp\left(\frac{-t-t_0}{\tau_1}\right)+C_0\right],$$

and bi-exponential,

$$M(t) = \frac{1}{2}\left[erf\left(\frac{t-t_0}{\sigma}\right)+1\right]\cdot\left[Aexp\left(\frac{-t-t_0}{\tau_1}\right)+Bexp\left(\frac{-t-t_0}{\tau_2}\right)+C_0\right],$$

fitting functions are used. More in detail, for the cut at the MC $\pi-\pi^*$ transition (2.15 eV, orange curve in Figure 5 a), a bi-exponential fit is performed, thus reflecting the fast shift induced by vibrational relaxation of MC molecules (captured by $\tau_2$), followed by the slow transition of excited MC molecules to their ground state (captured by $\tau_1$). For the cut at the Franck-Condon minimum of the MC excited state (1.85 eV, blue curve), a single-exponential fit is performed, reflecting only the transition of excited MC molecules to their ground state. We note that a slightly slower rise of the curve compared to the previous case can be observed (increase in $\sigma$) which is due to the vibrational relaxation of the excited MC molecules and subsequent shift of the stimulated emission towards the Franck-Condon minimum. In the following, the obtained time constant $\tau_1$ serves as a reference for the transition time of excited MC molecules to their ground state. In order to separate this relaxation of uncoupled MC molecules from dynamics induced by coupling with the Al antennas, we subtract a single-exponential decay with time constant $\tau_1$ from the time-traces of the coupled system. Raw data (grey) and data with the curve subtracted (blue) are shown in Figure 5 b in the main manuscript. Indeed, the subtraction leaves only a single-exponential decay that follows from interaction with the plasmons and can be fitted using a single-exponential function, now reflecting the time constant of the plasmonic-induced decay. The same subtraction is performed in Figure 5 c in the main manuscript, again leaving only the component induced by coupling with the plasmons. Raw data of Figure 5 c is shown in Supplementary Figure 21.

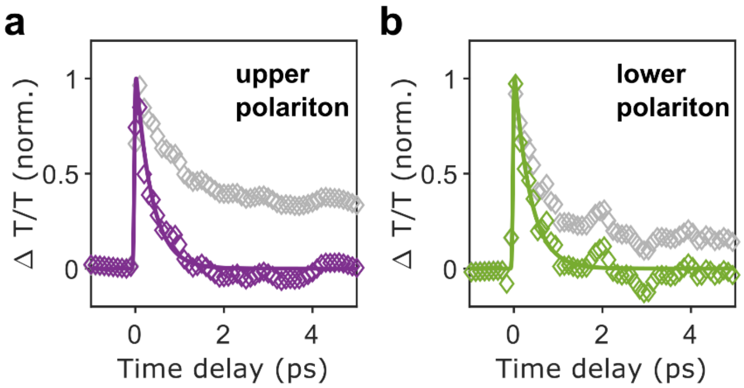


***Supplementary Figure 21:*** *$\Delta T/T$ at spectral position of upper (a) and lower (b) polariton states, respectively. Grey is raw data and colored is the data after subtraction of the slow decay component, as shown in main manuscript Figure 5c.*

## Supplementary References


**[1]** Fredriksson, H. et al. Hole–Mask Colloidal Lithography. *Advanced Materials* 19, 4297-4302 (2007).
**[2]** Remon, P. et al. Molecular Implementation of Sequential and Reversible Logic Through Photochromic Energy Transfer Switching. *Chem. Eur. J.* 17, 6492 – 6500 (2011).
**[3]** Görner, H. Photochromism of nitrospiropyrans: effects of structure, solvent and temperature. *Physical Chemistry Chemical Physics*, *3*(3), 416-423 (2001).
**[4]** Gaussian 16, Revision B.01, Frisch, M. J et al. J. Gaussian, Inc., Wallingford CT, 2016.
**[5]** Tomasi, J., Mennucci, B. & Cammi, R. Quantum Mechanical Continuum Solvation Models. *Chemical Reviews* 105, 2999-3094 (2005).
**[6]** Geuzaine, C. & Remacle, J. Gmsh: A 3-D finite element mesh generator with built-in pre- and post-processing facilities. *International Journal for Numerical Methods in Engineering* 79, 1309-1331 (2009).
**[7]** Fregoni, J. et al. Strong Coupling between Localized Surface Plasmons and Molecules by Coupled Cluster Theory. *Nano Letters* 21, 6664-6670 (2021).
**[8]** Mennucci, B. & Corni, S. Multiscale modelling of photoinduced processes in composite systems. *Nature Reviews Chemistry* 3, 315-330 (2019).
**[9]** Corni, S., Pipolo, S. & Cammi, R. Equation of Motion for the Solvent Polarization Apparent Charges in the Polarizable Continuum Model: Application to Real-Time TDDFT. *The Journal of Physical Chemistry A* 119, 5405-5416 (2014).
**[10]** Felicetti, S. et al. Photoprotecting Uracil by Coupling with Lossy Nanocavities. *The Journal of Physical Chemistry Letters* 11, 8810-8818 (2020).
**[11]** Antoniou, P., Suchanek, F., Varner, J. & Foley, J. Role of Cavity Losses on Nonadiabatic Couplings and Dynamics in Polaritonic Chemistry. *The Journal of Physical Chemistry Letters* 11, 9063-9069 (2020).
**[12]** Finkelstein-Shapiro, D. et al. Understanding radiative transitions and relaxation pathways in plexcitons. *Chem* 7, 1092-1107 (2021).
**[13]** Scholes, G. Limits of exciton delocalization in molecular aggregates. *Faraday Discussions.* 221, 265-280 (2019)
**[14]** S. Mukamel, Principles of nonlinear optical spectroscopy (Oxford University Press, 1995).
**[15]** Dovzhenko D. S. et al. Light–matter interaction in the strong coupling regime: configurations, conditions, and applications. *Nanoscale,* 10, 3589-3605 (2018).
**[16]** Gupta, N. S. et al. Complex plasmon-exciton dynamics revealed through quantum dot light emission in a nanocavity. Nature Communications 12, 1310 (2021)
**[17]** DelPo, C. et al. Polariton Transitions in Femtosecond Transient Absorption Studies of Ultrastrong Light–Molecule Coupling. *The Journal of Physical Chemistry Letters* 11, 2667-2674 (2020).
**[18]** Hohenester, U. & Trügler, A. MNPBEM – A Matlab toolbox for the simulation of plasmonic nanoparticles. *Computer Physics Communications* 183, 370-381 (2012).
**[19]** McPeak, K. et al. Plasmonic Films Can Easily Be Better: Rules and Recipes. *ACS Photonics* 2, 326-333 (2015).
**[20]** Hohenester, U. et al. Interaction of Single Molecules With Metallic Nanoparticles. IEEE J Sel Top Quantum Electron, 14,1430-1440 (2008).
**[21]** Palfrey, S.L. et al. Coherent interactions in pump–probe absorption measurements: the effect of phase gratings. *J. Opt. Soc. Am. B* 2, 674-679 (1985)
**[22]** Rafal Klajn. Spiropyran-based dynamic materials. *Chemical Society Reviews*, 43(1):148–184 (2014).
